# Comparison of gizzard and intestinal microbiota of wild neotropical birds

**DOI:** 10.1371/journal.pone.0194857

**Published:** 2018-03-26

**Authors:** M. Alexandra García-Amado, Hakdong Shin, Virginia Sanz, Miguel Lentino, L. Margarita Martínez, Monica Contreras, Fabian Michelangeli, María Gloria Domínguez-Bello

**Affiliations:** 1 Laboratorio de Fisiología Gastrointestinal, Centro de Biofísica y Bioquímica, Instituto de Investigaciones Científicas (IVIC), Caracas, Venezuela; 2 Department of Food Science and Biotechnology, College of Life Science, Sejong University, Seoul, South Korea; 3 Laboratorio de Biología de Organismos, Centro de Ecología, Instituto de Investigaciones Científicas (IVIC), Caracas, Venezuela; 4 Colección Ornitológica Phelps, Apartado, Caracas, Venezuela; 5 New York University Schools of Medicine, New York, United States of America; Consejo Superior de Investigaciones Cientificas, SPAIN

## Abstract

Gut bacterial communities have been shown to be influenced by diet, host phylogeny and anatomy, but most of these studies have been done in captive animals. Here we compare the bacterial communities in the digestive tract of wild birds. We characterized the gizzard and intestinal microbiota among 8 wild Neotropical bird species, granivorous or frugivorous species of the orders Columbiformes and Passeriformes. We sequenced the V4 region of the *16S rRNA* gene in 94 collected samples from 32 wild birds from 5 localities, and compared bacterial communities by foraging guild, organ, locality and bird taxonomy. *16S rRNA* gene-based sequencing data were examined using QIIME with linear discriminant analysis effect size (LEfSe) and metabolic pathways were predicted using PICRUSt algorism. We identified 8 bacterial phyla, dominated by Firmicutes, Actinobacteria and Proteobacteria. Beta diversity analyses indicated significant separation of gut communities by bird orders (Columbiformes vs. Passerifomes) and between bird species (*p*<0.01). In lower intestine, PICRUSt shows a predominance of carbohydrate metabolism in granivorous birds and xenobiotics biodegradation pathways in frugivorous birds. Gizzard microbiota was significantly richer in granivorous, in relation to frugivorous birds (Chao 1; non-parametric t-test, *p*<0.05), suggesting a microbial gizzard function, beyond grinding food. The results suggest that the most important factor separating the bacterial community structure was bird taxonomy, followed by foraging guild. However, variation between localities is also likely to be important, but this could not been assessed with our study design.

## Introduction

Birds have complex and unique diets, physiological traits, and developmental strategies. Additionally, flight capacity has been a strong selective pressure on many aspects of bird’s physiology, including the structure of their digestive tract, dietary adaptations and gut microbiota [1].

The study of microbial diversity and function in the avian gut has been focused mostly on captive, commercial species (chickens and turkeys), finding that gut microbiota changes with the diet, organ and age, and are influenced by factors such as captivity, antibiotic treatment, or pathogen colonization [2,3]. However, in recent years a number of studies have been done on wild birds such as hoatzin, vultures, kakapo, penguins, house sparrows, anseriformes and some neotropical and temperate birds [2,4–8]. These studies show that diet affects microbiota composition in birds. The predominant bacteria in the gut and feces of birds belong to the phyla Firmicutes, Actinobacteria, Bacteroidetes, and with variations in their relative proportions between herbivorous, carnivorous and omnivorous birds [5,9]. A previous study comparing the microbiota in gut organs between cows and hoatzin, a folivorous bird, concluded that organ function best explained the structure of the microbiota [10]. Recent studies have addressed the relationship between host taxonomy or geographical locations and gut microbiota in mammals and birds [2,5,6,8,9,11,12]

Studies of the intestine and gizzard microbiota have been focused on domestic birds [4,5,13,14] and the objective of this study was to characterize the microbiota in these organs across host taxonomy in neotropical wild birds with two different foraging guilds (granivory and frugivory).

## Materials and methods

### Animals

A total of 32 wild birds were captured using mist-nets in 5 different localities in Venezuela (**S1 Table**). The Venezuelan Environmental Ministry (N° 328) and IVIC bioethical animal commission (Dir-0884/1517/2014) approved the study.

Eight bird species from two orders (Columbiformes and Passeriformes) were classified according to their reported foraging guilds (granivores and frugivores). Consumption of insects have been only reported in the diet of Passeriformes studied [15]. Three species of granivorous birds were sampled: Ruddy Ground-Dove (*Columbina talpacoti*, n = 6), Common Ground-Dove (*Columbina passerina*, n = 10) and Rufous-collared Sparrow (*Zonotrichia capensis*, n = 2). There were 5 species of frugivorous birds including: Silver-beaked Tanager (*Ramphocelus carbo*, n = 3), Palm Tanager (*Thraupis palmarum*, n = 1), Glaucous Tanager (*Thraupis glaucocolpa*, n = 3), Lance-tailed Manakin (*Chiroxiphia lanceolata*, n = 3) and Buff-throated Saltator (*Saltator maximus*, n = 4).

### Samples

Birds were euthanized and kept frozen at -20°C until arrival to the laboratory, where the digestive organs (gizzard and intestine) were dissected and their contents collected with sterile cotton swabs to perform DNA extraction. The intestine was divided in two segments (upper section -the section contiguous to the stomach and lower section -the final section closer to cloacae).

A total of 96 samples were collected from 32 wild birds (18 granivorous and 14 frugivorous birds). Two samples were omitted for further analysis due to insufficient sequence coverage, yielding a total of 94 samples, including 32 gizzards, 31 upper and 31 lower intestines.

### *16S rRNA* gene-based sequencing analysis

DNA was extracted from samples from gizzard and intestine contents, using the Power Soil DNA Isolation Kit (MO BIO Laboratories, Carlsbad, CA, USA). The V4 region of the *16S rRNA* gene was amplified with barcode primers and sequenced as previously described [16]. Amplified DNA was sequenced using the Illumina HiSeq platform.

De-multiplexing *16S rRNA* gene sequences, quality control, chimeric sequence elimination, followed by closed-reference operational taxonomic units (OTUs) picking process at ≤ 97% sequence identity against the Greengenes database (13_8) was performed using the open source pipeline Quantitative Insights Into Microbial Ecology (QIIME) version 1.8.0 [17]. The total number of sequence reads was 5,828,311 (an average of 60,712 reads per sample). Each organ was analyzed independently and the total number of sequence reads in gizzard, upper and lower intestine were 2,081,059 (an average of 65,033 reads per sample), 1,867,516 (58,360 reads) and 1,879,736 (58,742 reads) respectively (paired-end, Phred> = Q20) (**S2 Table**).

Samples were rarefied to 14,077 reads per sample, and Chao 1 richness was used to estimate the alpha diversity comparing foraging guild in each organ separately. Tukey’s post-hoc test was used to determine significant differences. Weighted and unweighted UniFrac distances were used to evaluate beta diversity (diversity between groups) with principal coordinate analysis (PCoA).

Phylogenetic Investigation of Communities by Reconstruction of Unobserved States (PICRUSt) was used to predict the metabolic pathways from *16S rRNA* gene-based microbiota of gizzard and intestine of birds studied [18]. The abundance of each OTU was normalized using the Greengenes database version 13_5. The predicted functions (KOs) were then collapsed into hierarchical KEGG (Kyoto Encyclopedia of Genes and Genomes) pathways using the categorize_by_function step in the PICRUSt pipeline.

### Statistical analyses

A core microbiota analysis was also run in QIIME to identify OTUs (bacterial taxa) present in >90% of the samples included in this study. The goal of this analysis is to identify taxa that may be shared across digestive tract of bird species [19].

We used the statistical analysis of similarity (ANOSIM) [20] and the non-parametric multivariate ANOVA based on dissimilarities (Adonis) with 999 random permutations [21] implemented in QIIME to determine the relationship between categorical variables associated with each bird and the microbial communities. The categorical variables tested were foraging guild, sampling locality and bird taxonomy (order and species) in each organ. The ANOSIM statistic compares the mean of ranked dissimilarities between groups to the mean of ranked dissimilarities within groups. An R value close to "1.0" suggests dissimilarity between groups while an R value close to "0" suggests an even distribution of high and low ranks within and between groups [22]. Adonis is a function for the analysis and partitioning of sums of squares using semimetric and metric distance matrices. Adonis returns a p value for significance, and an R^2^ value, indicative of the amount of variation explained by a specific variable [21]. A linear multivariate regression model (MaAsLin, Multivariate microbial Association by Linear models) was applied to determine significant associations between microbiota and categorical variables associated with each bird (foraging guild, organ, locality and bird taxonomy) [23].

Linear discriminant analysis (LDA) effect size (LEfSe) was calculated to identify bacterial lineages whose frequencies differed significantly as a function of foraging guild and bird taxonomic variables in each organ. LEfSe detects differentially distributed lineages with the Kruskall-Wallis test, and checks the consistency of subclass distinctions with the pairwise Wilcoxon text. The final linear discriminant analysis was used to rank all differentiating lineages by their effect size. LEfSe was used with default parameters on species-level OTU tables (operational taxonomical unit) to determine taxa that best characterized each population; only features with LDA score >3.0 were kept [24].

MaAsLin, LEfSe and PICRUSt analyses were completed using the Galaxy platform (http://huttenhower.sph.harvard.edu/galaxy/).

## Results

The digestive tract of the birds studied share a core microbiota (**S3 Table**). All gizzards shared 40 OTUs, belonging to the phyla Bacteroidetes, Cyanobacteria, Planctomycetes, Verrucomicrobia and Alpha and Gammaproteobacteria. Ninety percent of upper and lower intestines shared a total of 58 and 36 OTUs respectively. The phylum Proteobacteria had the largest number of OTUs shared in all digestive tracts. Common families of shared Proteobacteria OTUs included Hyphomonadaceae (Class Alphaproteobacteria) and Thiohalorhabdales (Class Gammaproteobacteria).

**Fig 1:**
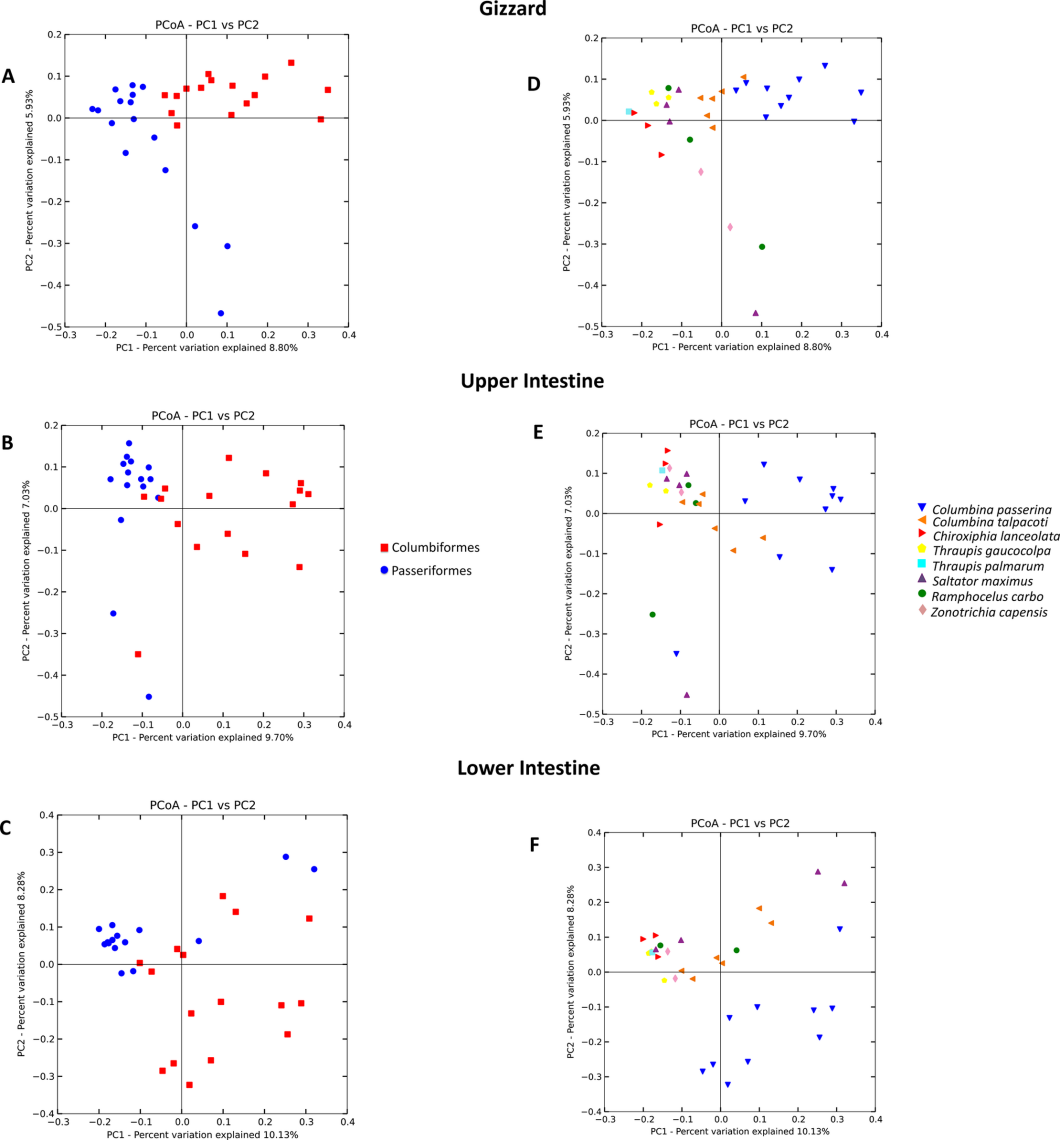
Beta diversity of gut bacterial communities by bird taxonomy in different organs. A, B, C) Principal coordinates analysis (PCoA) of unweighted UniFrac distances of bacterial communities by bird orders (Columbiformes and Passeriformes). D, E, F) PCoA of unweighted UniFrac distances of bacterial communities by bird species.

**Fig 2:**
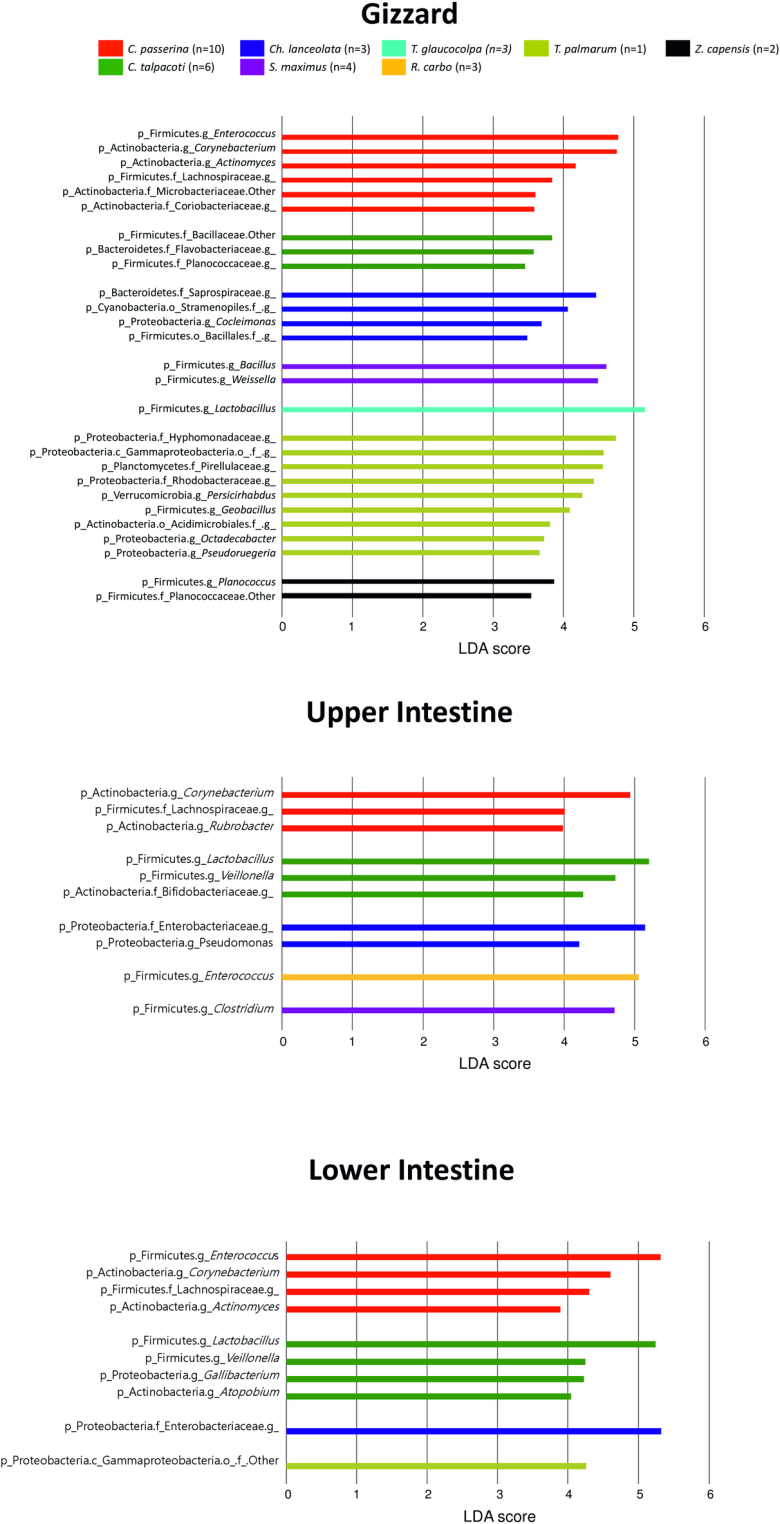
Linear discriminatory analyses (LEfSe) of bacterial taxa discriminant of bird species (LDA>3). Histogram showing abundance of OTUs according to bird species in the gizzard, upper and lower intestine (Granivorous: *C*. *passerina*, *C*. *talpacoti* and *Z*. *capensis*. Frugivorous: *Ch*. *lanceolata*, *R*. *carbo*, *S*. *maximus*, *T*. *glaucocolpa* and *T*. *palmarum*).

Beta diversity analyses indicated significant separation of gut communities by host taxonomic categories in each organ studied (*p*< 0.05; **Fig 1, S1 Fig, S4 Table**). LEfSe and Multivariate Association with Linear Models (MaAsLin) analyses showed that bird taxonomy (species) showed the highest number of significant associations with microbial taxa, particularly in the gizzard (**Fig 2, S5 Table**).

**Fig 3:**
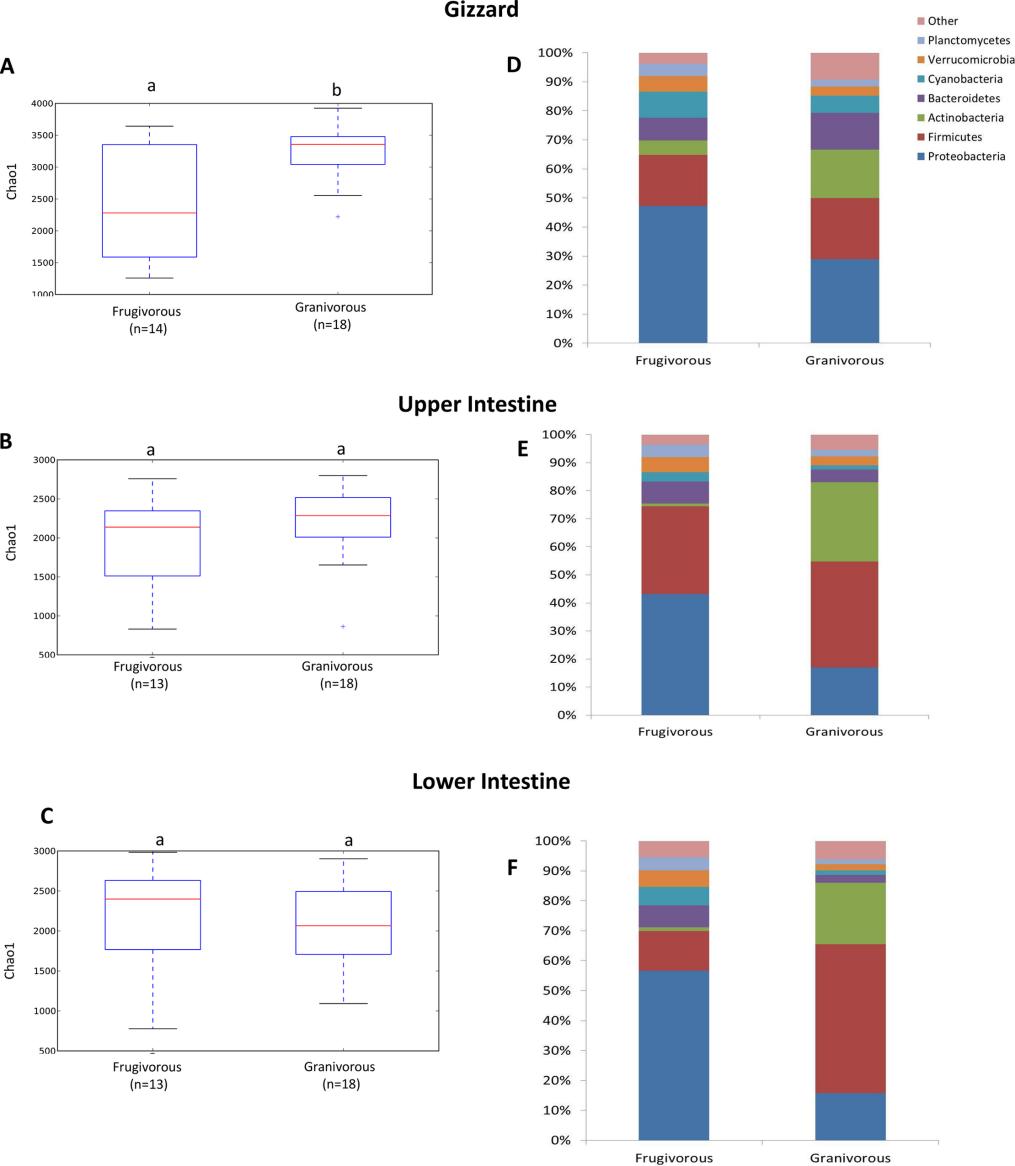
Alpha diversity of gut bacterial communities by frugivorous and granivorous birds in different organs. A, B and C) Bacterial community richness (Chao1 index) for the gizzard, upper and lower intestine. D, E and F) Relative abundances of phyla (%) present in the gizzard, upper and lower intestine. Different letters above boxplots indicate significant differences (non-parametric t-test ≤0.05).

Granivorous birds showed higher gizzard bacterial alpha diversity, in relation to frugivorous birds (Chao 1 for richness; non-parametric t-test, *p*< 0.05; **Fig 3A**). However, there were no significant differences in bacterial alpha diversity by foraging guild in upper or lower intestine (**Fig 3B and 3C**). Granivorous birds gizzards were characterized by bacterial communities dominated by Proteobacteria (29%), Firmicutes (21%), and Actinobacteria (17%) whereas that of frugivorous birds had a higher proportion of Proteobacteria (47%; **Fig 3D**). In the intestines, frugivorous birds had an absolute dominance of Proteobacteria (43% in upper and 57% in lower intestines), while in granivorous birds the intestine was dominated by Firmicutes (38% in upper and 50% in lower) and Actinobacterias (28% in upper and 21% in lower; **Fig 3E and 3F**).

**Fig 4:**
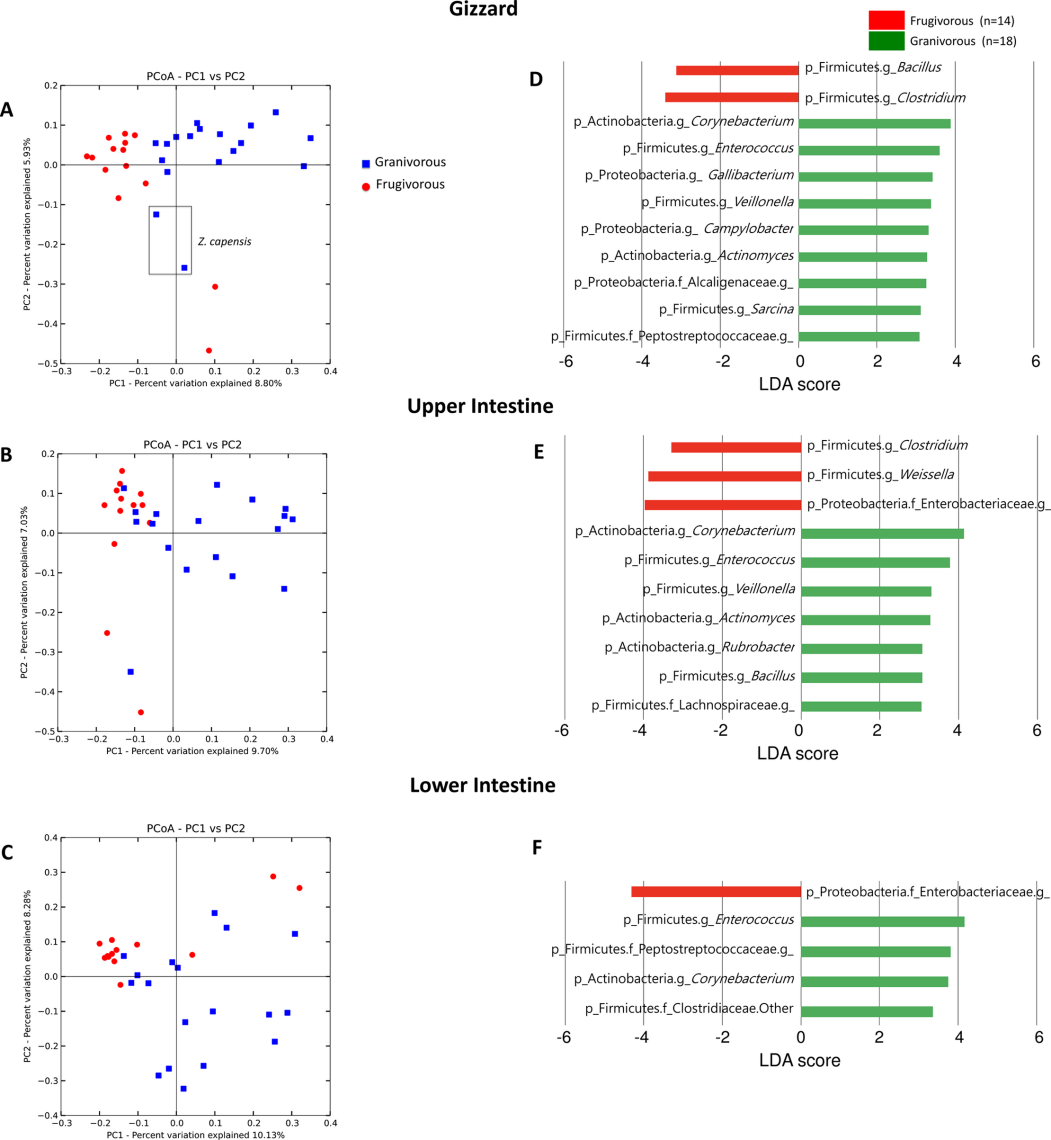
Beta diversity of bacterial communities in different organs of frugivorous and granivorous birds. A, B, C) Principal coordinates analysis (PCoA) of unweighted UniFrac distances. D, E F) Linear discriminatory analyses (LEfSe) of bacterial taxa discriminant of frugivorous and granivorous birds (LDA>3). Histogram showing OTUs that are more abundant in granivorous and frugivorous birds by gizzard, upper and lower intestine.

We observed significant separation of bacterial beta diversity between gizzards of granivorous and frugivorous birds (ANOSIM R = 0.43, *p*< 0.05; **Fig 4A, S2 Fig, S4 Table**). However there was no significant difference in bacterial beta diversity by foraging guilds in upper or lower intestine (**Fig 4B and 4C, S2 Fig, S4 Table**). Adonis statistical test showed significant differences in all organs (*p*< 0.05) but R^2^ values were low; indicating that only 6–7% of interspecific differences can be explained by foraging guilds. We observed significant associations of specific microbial taxa (n = 6) with foraging guilds using MaAsLin analysis (**S5 Table**).

To identify specific bacterial taxa differentially distributed between granivorous and frugivorous birds in each organ, we performed LEfSe analysis (LDA score> 3.0; **Fig 4D, 4E and 4F**). In the gizzard, a total of 11 genera were differentially represented among the two foraging guilds, with 8 genera (*Corynebacterium*, *Enterococcus*, *Gallibacterium*, *Veillonella*, *Campylobacter*, *Actinomyces*, *Sarcina*, and two unidentified genera in Peptostreptococcaceae and Alcaligenaceae families) being more abundant in granivorous birds and two genera (*Bacillus* and *Clostridium*) in frugivorous birds. In the upper intestine, *Corynebacterium*, *Enterococcus*, *Veillonella*, *Actinomyces*, *Rubrobacter*, *Bacillus*, and an unidentified genus in Lachnospiraceae family were overrepresented and *Clostridium*, *Weissella*, and an unidentified genus in Enterobacteriaceae family were depleted in granivorous birds, in relation to frugivorous birds. In lower intestine, *Enterococcus*, *Corynebacterium*, unidentified genera in Peptostreptococcaceae and Clostridiaceae were overrepresented and an unidentified genus in Enterobacteriaceae was depleted in granivorous birds, in relation to frugivorous birds. *Corynebacterium* (Phylum Actinobacteria) and *Enterococcus* (phylum Firmicutes) are overrepresented in granivorous birds among all organs (**Fig 4D, 4E and 4F**).

The microbiota of *Z*. *capensis*, is more related to other Passeriformes despite its granivorous diet, than to other granivores from the Columbiformes (**Fig 1D, 1E and 1F**). Amongst granivores, ANOSIM analyses show differences only in the gizzard of Z. *capensis* and *C*. *passerina* and the upper intestine of Z. *capensis* and *C*. *talpacoti* (*p*< 0.05; **S4 Table**). Adonis analyses show significant differences in all organs of Z. *capensis* and *C*. *passerina* and in the gizzard and upper intestine of Z. *capensis* and *C*. *talpacoti* (*p*< 0.05; **S4 Table**).

**Fig 5:**
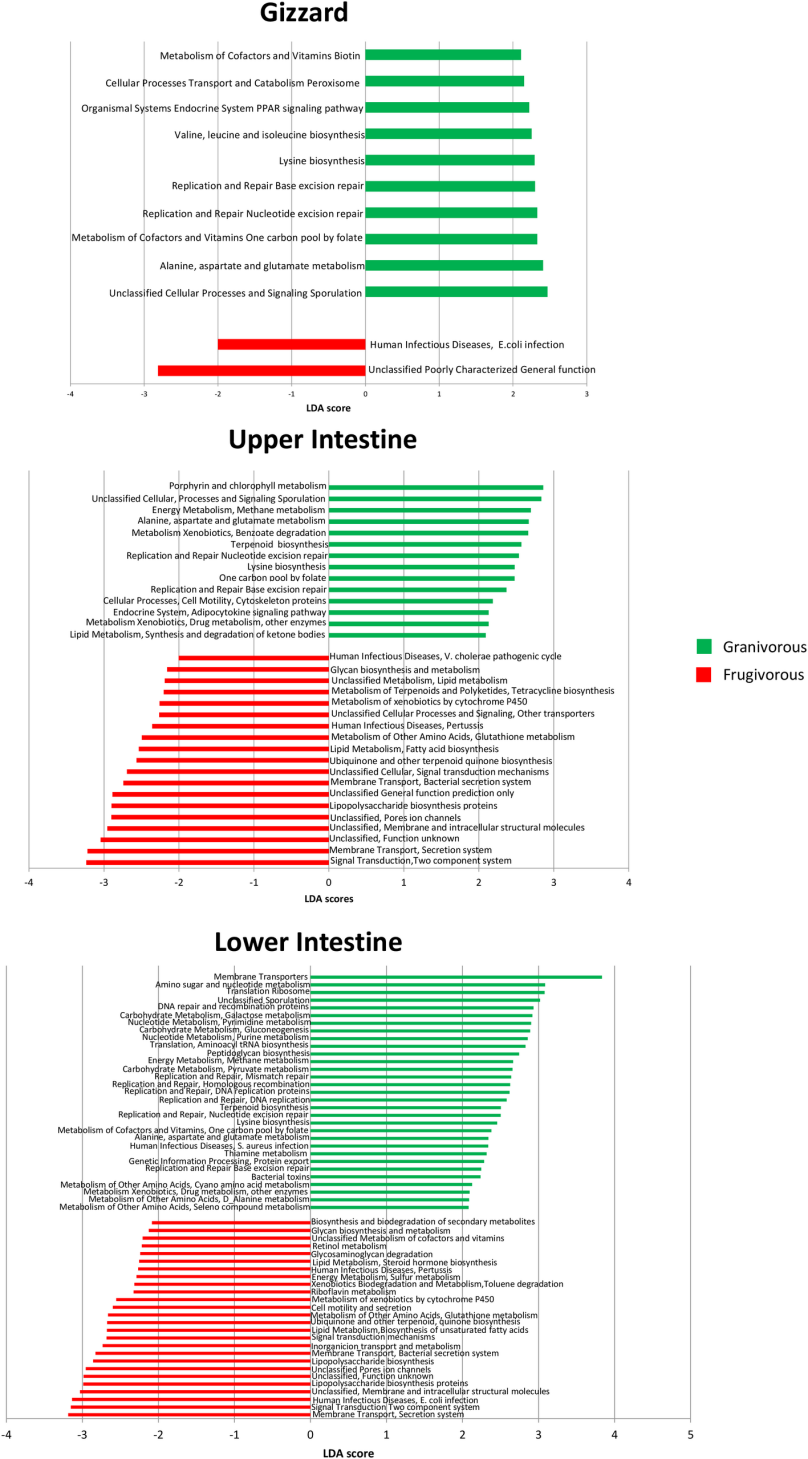
Linear discriminatory analyses (LEfSe) comparing KEGG module predictions using PICRUSt in different organs from granivorous and frugivorous birds (LDA>2).

Microbial metabolic pathways were estimated based on the *16S rRNA* gene data using the PICRUSt software and comparisons between granivorous and frugivorous birds in each organ were made using LEfSe (**Fig 5**). Predicted functional analysis showed differences in KEGG pathways in each organ when comparing granivorous and frugivorous birds, in particular in the lower intestine. In granivores, aminoacid and vitamins metabolism pathways were abundant in gizzards and upper intestines, and energy and carbohydrate metabolism predominate in the lower intestine. Lipid metabolism and xenobiotics biodegradation are overrepresented in intestines of frugivorous birds.

ANOSIM and Adonis analyses show weak significant differences in most comparisons (**S4 Table**). ANOSIM test show that most variations in the gut microbiota are explained by the host taxonomic categories and foraging guild (p<0.05, R: 0.33–0.58). However, Adonis shows that categorical variables that explain the highest amount of variation are host taxonomy and locality (p<0.05, R^2^: 0.19–0.40). The PCoA using weighted and unweighted unifrac analyses for sampling localities were included, but we did not observe a differential pattern between localities (**S3 Fig**). However, MaAsLin analyses show that sampling localities (Ramal de Calderas and Sierra de Tirgua) had significant associations to specific microbial taxa (n = 14) (**S5 Table**). The interpretation of these results is difficult because bird taxonomy overlapped with foraging guild and most of birds’ species were captured in different localities. However to simplify the interpretation, we carried out PCoA analysis comparing two frugivorous bird species (*R*. *carbo* and *S*. *maximus*) in three localities (Ramal de Calderas, Uey river and Sierra de Tirgua) to determine the effect between birds taxonomy and locality (**S4 Fig**). We observed significant differences (ANOSIM R: 0.78; Adonis R^2^: 0.86; p <0.05) between localities only for the gizzards (**S4D Fig**).

Alpha and beta bacterial diversity of crops of Columbiformes birds showed a high proportion of Actinobacteria (45%) (**S5A Fig**), with no significant differences in alpha diversity, from the lower intestine (Chao1 index richness between organs t test, p = 0.07) (**S5B Fig**). Gizzard and crop shared 10.2% of OTUs and 13.6% of OTUs were unique in these organs (**S5D Fig**).

## Discussion

Studies about gut microbiota in wild birds have increased in recent years, demonstrating the importance of the role of microorganism in host physiology [2,4–8]. This study contributes to the bacterial characterization of gizzard and intestine from 8 species of wild birds with two different foraging guilds in five different localities.

Studies in temperate and neotropical birds showed that gut microbiota variation is related to bird taxonomy [6,8]. However, several avian studies showed that diet is an important factor to determine the microbiota composition [5]. Furthermore, other studies in mammals, one on the fecal microbiota of 60 mammalian species, and another in Phyllostomidae bats demonstrated that the gut microbiome composition was intimately related to host-phylogeny and feeding-strategies [9,25]. In our study, PCoA, ANOSIM, Adonis, MaAsLin and LEfSe analyses show that microbiota variation is mainly explained by bird taxonomy and locality (**Figs 1 and 2, S4 and S5 Tables**).

The bird taxonomy and foraging guild were closely related as the granivorous birds belonged to the Columbiformes order and the frugivorous birds belonged to the Passeriformes order, except for *Z*. *capensis*, which belongs to the Passeriformes but is a seed eater. However, ANOSIM and Adonis analyses show that microbiota differences were more significant between Columbiformes vs. Passeriformes birds than between granivorous vs frugivorous foraging guilds (**S4 Table**), as had previously been reported in other temperate and neotropical birds [6,8].

In this study, alpha diversity was higher in gizzards from granivorous than from frugivorous birds (**Fig 3A**) and ANOSIM analysis showed significant beta diversity differences (**S4 Table**). Firmicutes, Actinobacteria and Proteobacteria were the phyla most commonly found in granivorous birds (**Fig 3D, 3E and 3F**). Similar results have been found in other seed eating birds like Northern Bobwhite (*Colinus virginianus*) [26], House Sparrow (*Passer domesticus*) [4] and chicken [2]. Starches are the most abundant carbohydrates in seeds. These complex polysaccharides are difficult to digest for vertebrates [27]. The rich bacterial community in granivorous birds is probably associated to the transformation of complex carbohydrates to short-chain fatty acids (SCFAs) mediated by consortia of multiple bacteria with complementary capabilities [27]. This observation is supported by PICRUSt analyses, where carbohydrate metabolism is predominant in the lower intestine of granivorous birds (**Fig 5**).

LEfSe analyses showed that the Clostridiaceae family (Firmicutes phylum) is abundant in the lower intestine of granivorous birds and *Clostridium* genus is predominant in the gizzard and upper intestine in frugivorous birds (**Fig 4D, 4E and 4F**), particularly in Buff-throated Saltator (**Fig 2**). This family of bacteria is abundant in the ceca of granivorous chicken [13,28], neotropical birds [6], gastrointestinal tract of turkeys [29] and in vulture facial skin and hindgut samples [14]. The members of this bacterial family are associated with proteolytic activity and complex carbohydrate degradation, [30–32]. It is possible that the predominance of amino acid and vitamin metabolism pathways (**Fig 5**) found in the intestine of studied birds could be associated to this family.

Firmicutes, Actinobacteria, Bacteroidetes, and Proteobacteria are the predominant phyla found in the avian gut microbiota with carnivore (penguins and vultures) and herbivore diets (kakapo, turkey, duck, goose and emu) [2]. Bacteroidetes are known for their ability to degrade complex carbohydrates, including starch [33]. However, in our study only 2–13% of OTUs found belong to this phylum. Low proportion of Bacteroidetes has been reported for Northern Bobwhite (0.02%) [26], Parrots (0.2%) [34], pet birds (0.03%) [35] and different orders of temperate (2.7%) and neotropical birds [6,8]. The significance of low proportion of Bacteroidetes species in certain birds is unknown.

A large proportion of Proteobacteria was detected in all organs of frugivorous birds (between 43–57%) (**Fig 3D, 3E and 3F**). This is higher than that found in the gut of some mammal species [36]. Nevertheless, similar results have been reported in other wild birds with herbivorous, frugivorous and omnivorous dietary habits [2,6,8,37] and in wild bats with frugivorous diets [38]. Wang and coauthors [37], hypothesized that high Proteobacteria abundance in the gut of wild birds with frugivorous diets may significantly contribute to increase digestive efficiency and assimilation, which may play an important role in providing energy and nutrients. Additionally, the low content of nitrogen in fruits could be favoring nitrogen-fixing bacteria that use atmospheric nitrogen to produce ammonium, which has been reported in diverse taxonomical groups of bacteria [39]. Nitrogen-fixing bacteria belonging to Gammaproteobacteria and Alphaproteobacteria were reported before in termites [40] and Amazonian catfish (*Panaque nigrolineatus*) [41]. Nitrogenase activity has been reported in herbivorous grouses [42]. In this study, LEfSe analyses show Gammaproteobacteria (Enterobacteriaceae family) predominance in upper and lower intestines of frugivores and this might be associated with nitrogen-fixing bacteria; however more studies are necessary to demonstrate their presence.

Comparing the bacterial community by organ, we observed that gizzards from granivorous birds possessed a bacterial community significantly richer than frugivorous birds (**Fig 3A**). Additionally, LEfSe analyses show that the gizzard of granivorous birds had a higher diversity of bacterial taxa than the intestine of granivorous birds (**Fig 4D, 4E and 4F**). It has been reported in chicken that the gizzard functions to grind food, and similar microbial communities were found in the crop and gizzard [43]. In this study, well developed crops were observed only in Columbiformes birds. Therefore, the crop was not included in the analyses; however, alpha and beta bacterial diversity of Columbiformes including crops were made (**S4 Fig**).

Crop and gizzard of Columbiformes had no significant differences in the Chao1 index richness or PCoA analysis (**S4B and S4C Fig**). This suggests that most bacteria found in gizzards were also present in crops. However, the distribution of OTUs at the phylum level shows that the gizzard had a higher proportion of Proteobacteria and Bacteroidetes and that 13.6% of OTUs were unique, suggesting that gizzard function is more than grinding food (**S4A and S4D Fig**) and may imply that different environmental conditions promote the establishment of these bacterial phyla.

Frugivorous birds have a small gizzard and short intestine with a higher rate of food passage. This is an adaptation to digest food with high proportion of simple carbohydrates and water, and low content of protein and fiber as fruit pulp [44,45]. This may suggest that microorganisms have little contribution in nutrient assimilation [45]. In our study, frugivorous birds had small, non-muscular gizzards and short intestines and their bacterial community had few differences between organs. The high proportion of easily digestible carbohydrates in their diets could explain these few differences observed in bacterial communities between organs. On the other hand, the microbiota of these birds might be associated to nitrogen metabolism. Some nectarivorous, omnivorous, granivorous and herbivorous birds reflux uric acid into the intestine and ceca where bacteria degrade the uric acid for amino acid synthesis that, in turn birds can reabsorb [46,47]. Additionally, PICRUSt analysis shows a predominance of xenobiotics biodegradation pathways suggesting that these bacteria could help in other functions such as detoxification of plant secondary compounds, synthesis of vitamins, prevention of pathogen colonization and regulation of the immune system [32,48].

Recent studies demonstrated that factors, such as locality and habitat, are important in the microbial structure, because variables like local flora and fauna, photoperiod, available food, climate conditions, etc. may affect host microbiota [11,12]. Our study is not suitable to fully assess the importance of the locality variable in shaping the microbiota, because our 8 bird species were captured in 5 different localities. However, to determine the effect on microbiota between birds’ taxonomy and locality, we compare two frugivorous bird species (*R*. *carbo* and *S*. *maximus*) in three localities (Ramal de Calderas, Uey river and Sierra de Tirgua) **(S4 Fig**). In gizzards, the PCoA analysis shows that host microbiota is more related to the locality (Ramal de Calderas) than to the species identity (**S4A and S4D Fig**), suggesting that host environment had an important effect on microbiota composition. Therefore, we cannot discard the importance of locality in the composition of microbial community of the host digestive tract. Future studies should address the host microbiota variations by locality in detail.

## Conclusion

The results of this study indicate that host taxonomy and foraging guild are strong modulators of the gut bacterial community structure; gizzard bacteria were more diverse than the intestinal bacteria, in granivorous but not in frugivorous birds. Additionally, variation between localities is also likely to be important, but this could not be adequately assessed with our study design.

## Supporting information

S1 Fig Beta diversity of bacterial communities by bird taxonomy in different organs. A, B, C) Principal coordinates analysis (PCoA) in Columbiformes and Passeriformes birds. D, E, F) PCoA in bird species. Weighted UniFrac distances.
(RAR)

S2 Fig Beta diversity of bacterial communities in different organs from frugivorous and granivorous birds. Weighted UniFrac distances.
(RAR)

S3 Fig Beta diversity of bacterial communities in different organs from different sampling localities. A, B, C) Unweighted UniFrac distances. D, E, F) Weighted UniFrac distances.
(RAR)

S4 Fig Beta diversity of bacterial communities by bird taxonomy in different organs from different sampling localities. A, B, C) Principal coordinates analysis (PCoA) of *R*. *carbo* and *S*. *maximus*. D, E, F) PCoA in sampling localities. Weighted UniFrac distances.
(RAR)

S5 Fig Alpha and Beta diversity in digestive organs of Columbiformes birds. A) Relative proportion of different bacterial phyla. B) Bacterial community richness (Chao1 index). C) Principal coordinates analysis of unweighted UniFrac distances. D) Venn diagram from OTU table generated by QIIME to illustrate the number of shared and unique OTUs between all organs. Venn diagram was made using the program: http://bioinfogp.cnb.csic.es/tools/venny/index.html
(RAR)

S1 Table Order, family, species, foraging guild, habitat, elevation, sampling locality, and number of birds individuals used in this study. (DOCX)

S2 Table Summary information of V4 *16S rRNA* sequences analyses. (XLSX)

S3 Table Core OTUs present in ≥90% of the samples. (XLSX)

S4 Table Statistical significance of comparisons of bacterial communities from different organs, foraging guild, locality or host taxonomy. ANOSIM of gut bacterial abundance data was used to generate a permutated Global R statistic (R) and permutated p-value (p). Significance level: **R ≥0.5,*R = 0.3 to 0.5. R^2^ values of Adonis test for significance across the weighted and unweighted UniFrac distance matrices. Significance level: ** p-values ≤ 0.01, * p-values ≤ 0.05.
(DOCX)

S5 Table List of significant associations between microbiota and categorical variables associated with each bird (foraging guild, sampling locality and bird taxonomic variables) using a Multivariate Microbial Association by Linear models (MaAsLin). (XLSX)
