# Supplementary material for: Comparison of gizzard and intestinal microbiota of wild neotropical birds
Source: PLoS One. 2018 Mar 26;13(3):e0194857. doi: 10.1371/journal.pone.0194857 (PMC5868825; doi:10.1371/journal.pone.0194857)
Supplement: S1 Table — (DOCX) [file pone.0194857.s006.docx]

| Order | Family | Specie | N | Diet | Habitat | Elevation  (m) | Margarita Island | Caracas | Sierra de Tirgua | Ramal de Calderas | Uey River |
| --- | --- | --- | --- | --- | --- | --- | --- | --- | --- | --- | --- |
| Columbiformes | Columbidae | *Columbina talpacoti* | 6 | Granivorous | Urban area | 1100 |  | 6 |  |  |  |
| Columbiformes | Columbidae | *Columbina passerina* | 10 | Granivorous | Arid scrub | 100 | 10 |  |  |  |  |
| Paseriformes | Thraupidae | *Rhamphoceluscarbo* | 3 | Frugivorous | Stubble  Scrubland | 123  1250 |  |  |  | 1 | 2 |
| Paseriformes | Cardinalidae | *Saltator maximus* | 4 | Frugivorous | Coffee lots  Scrubland  secondary forest | 1250  1250  1350 |  |  | 2 | 1  1 |  |
| Paseriformes | Emberizidae | *Zonotrichia capensis* | 2 | Granivorous | Scrubland | 1250 |  |  |  | 2 |  |
| Paseriformes | Thraupidae | *Thraupis gaucocolpa* | 3 | Frugivorous | Aridscrub | 100 | 3 |  |  |  |  |
| Paseriformes | Thraupidae | *Thraupis palmarum* | 1 | Frugivorous | Aridscrub | 100 | 1 |  |  |  |  |
| Paseriformes | Pipridae | *Chiroxiphia lanceolata* | 3 | Frugivorous | Aridscrub | 100 | 3 |  |  |  |  |
