# Supplementary material for: Comparison of gizzard and intestinal microbiota of wild neotropical birds
Source: PLoS One. 2018 Mar 26;13(3):e0194857. doi: 10.1371/journal.pone.0194857 (PMC5868825; doi:10.1371/journal.pone.0194857)
Supplement: S4 Table — ANOSIM of gut bacterial abundance data was used to generate a permutated Global R statistic (R) and permutated p-value (p). Significance level: **R ≥0.5,*R = 0.3 to 0.5. R2 values of Adonis test for significance across the weighted and unweighted UniFrac distance matrices. Significance level: ** p-values ≤ 0.01, * p-values ≤ 0.05. (DOCX) [file pone.0194857.s009.docx]

| Source |  | Gizzard | | | | Upper intestine | | | | Lower Intestine | | | |
| --- | --- | --- | --- | --- | --- | --- | --- | --- | --- | --- | --- | --- | --- |
|  |  | ANOSIM | | Adonis | | ANOSIM | | Adonis | | ANOSIM | | Adonis | |
|  |  | R | p | R^2^ | p | R | p | R^2^ | p | R | p | R^2^ | p |
| Granivores vs Frugivores | unweighted | 0.43* | 0.01 | 0.07 | 0.001** | 0.28 | 0.01 | 0.06 | 0.002** | 0.28 | 0.01 | 0.06 | 0.001** |
|  | weighted | 0.22 | 0.01 | 0.09 | 0.007** | 0.22 | 0.02 | 0.14 | 0.002** | 0.07 | 0.12 | 0.09 | 0.025* |
| Locality | unweighted | 0.18 | 0.09 | 0.19 | 0.005** | 0.03 | 0.36 | 0.20 | 0.001** | 0.07 | 0.29 | 0.21 | 0.001** |
|  | weighted | 0.34* | 0.02 | 0.40 | 0.001** | 0.07 | 0.19 | 0.28 | 0.014* | 0.12 | 0.17 | 0.20 | 0.161 |
| Bird order | unweighted | 0.42* | 0.01 | 0.07 | 0.001** | 0.38* | 0.01 | 0.07 | 0.001** | 0.33* | 0.01 | 0.07 | 0.001** |
|  | weighted | 0.13 | 0.01 | 0.09 | 0.014* | 0.30* | 0.01 | 0.18 | 0.001** | 0.17 | 0.01 | 0.13 | 0.010* |
| Bird specie | unweighted | 0.58** | 0.01 | 0.29 | 0.001** | 0.37* | 0.01 | 0.29 | 0.001** | 0.36* | 0.01 | 0.30 | 0.001** |
|  | weighted | 0.38* | 0.01 | 0.41 | 0.002** | 0.24 | 0.04 | 0.40 | 0.004** | 0.06 | 0.32 | 0.30 | 0.081 |
| *Z. capensis* vs *C. passerina* | unweighted | 0.65** | 0.01 | 0.13 | 0.024* | 0.38 | 0.07 | 0.12 | 0.037* | 0.33 | 0.09 | 0.12 | 0.038* |
|  | weighted | 0.20 | 0.18 | 0.17 | 0.098 | 0.21 | 0.17 | 0.19 | 0.082 | 0.11 | 0.32 | 0.18 | 0.084 |
| *Z. capensis* vs *C. talpacoti* | unweighted | 0.85 | 0.05 | 0.19 | 0.032* | 0.75** | 0.01 | 0.18 | 0.042* | 0.48 | 0.06 | 0.17 | 0.077 |
|  | weighted | 0.47 | 0.16 | 0.28 | 0.031* | 0.46 | 0.07 | 0.36 | 0.035* | 0.02 | 0.54 | 0.21 | 0.165 |
